# Establishing the role of ZBTB20 mutations in GnRH deficiency and impaired neurogenesis in the subventricular zone: a human cohort and animal model study

**DOI:** 10.1016/j.ebiom.2026.106416

**Published:** 2026-08-04

**Authors:** Jun Guan, Jingtan Zhu, Yuting Li, Min Nie, Jing Zhang, Zhiheng Chen, Huadie Liu, Dan-Na Chen, Ruizhi Zheng, Meichao Men, Jia-Da Li

**Affiliations:** a Furong Laboratory, Center for Medical Genetics, School of Life Sciences, Central South University, Changsha, Hunan 410078, PR China; b Hunan Key Laboratory of Animal Models for Human Diseases, Changsha, Hunan 410078, PR China; c Hunan Key Laboratory of Medical Genetics, Changsha, Hunan 410078, PR China; d Medical Genetic Center, Jiangxi Maternal and Child Health Hospital, No 318, Bayi Avenue, Nanchang, China; e Department of Pediatrics, The Third Xiangya Hospital, Central South University, Changsha, Hunan 410013, China; f NHC Key Laboratory of Endocrinology, Department of Endocrinology, Peking Union Medical College Hospital, Peking Union Medical College, Chinese Academy of Medical Sciences, Beijing 100730, China; g Department of Laboratory Animals, Central South University, Changsha, Hunan 410078, China; h MOE Key Laboratory of Rare Pediatric Diseases, Hengyang Medical School, University of South China, Hengyang, Hunan 421001, PR China; i Department of Basic Medical Sciences, Changsha Medical University, Changsha, Hunan 410219, China; j Department of Endocrinology, The People’s Hospital of Henan Province, Zhengzhou, Henan 450003, China; k Department of Clinical Nutrition, Xiangya Hospital, Central South University, Changsha, Hunan 410078, China; l National Clinical Research Center for Geriatric Disorders, Xiangya Hospital, Central South University, Changsha, Hunan 410078, China

**Keywords:** Congenital hypogonadotropic hypogonadism, Kallmann syndrome, *ZBTB20*, Genetics

## Abstract

**Background:**

Congenital hypogonadotropic hypogonadism (CHH) arises from defective development or dysfunction of GnRH neurons. Olfactory bulb (OB) malformations frequently accompany CHH, a condition termed Kallmann syndrome (KS). Nevertheless, approximately 50% of CHH cases remain genetically undefined.

**Methods:**

Leveraging our 15-year effort in building one of Asia’s largest CHH cohorts (812 unrelated patients, and 49 CHH families), we identified a pathogenic *ZBTB20* mutation (p.R300C) in a four-generation CHH family with seven affected members. We generated nervous system-specific *Zbtb20* conditional knockout (cKO) mice to assess phenotypic and mechanistic consequences. Molecular analyses, including transcriptional regulation assays, were performed to explore downstream pathways.

**Findings:**

Four additional heterozygous *ZBTB20* variants were detected in the CHH cohort. *Zbtb20* cKO mice recapitulated hallmark CHH features: GnRH neuron deficiency, hypogonadism, and infertility. Notably, migration of GnRH neurons in *Zbtb20*-deficient embryos was stalled along the defective terminal nerve (TN) scaffold. Furthermore, *Zbtb20* deficiency impaired proliferation of neural stem cells (NSCs) in the subventricular zone (SVZ) and disrupted their migration via the rostral migratory stream (RMS) to the OB. We also identified *Thbs4* as a key downstream target, as ZBTB20 transcriptionally activates *Thbs4*, which is critical for SVZ NSCs migration.

**Interpretation:**

Our study establishes *ZBTB20* as a CHH-associated gene and demonstrates its essential role in SVZ-OB neurogenesis. Loss of *ZBTB20* function leads to OB atrophy, disrupted terminal nerves, GnRH deficiency and CHH pathogenesis.

**Funding:**

This project is financially supported by 10.13039/501100001809National Natural Science Foundation of China, and the 10.13039/501100004735Natural Science Foundation of Hunan Province.

**Research in context**

**Evidence before this study**

A systematic literature review was performed by searching Web of Science and PubMed using the following terms: (“ZBTB20”) AND (“Kallmann syndrome” OR “congenital hypogonadotropic hypogonadism” OR “GnRH deficiency”), with no language restrictions. Accumulating work has uncovered critical developmental events relevant to CHH pathogenesis, yet many genetic contributors remain to be discovered. The terminal nerve (TN) is thought to serve as a migratory scaffold for GnRH neurons. Olfactory bulb (OB) malformations are frequently observed in patients with CHH with anosmia/hyposmia, i.e., Kallmann syndrome (KS). Nevertheless, severe OB hypoplasia does not invariably lead to reproductive phenotypes, and the link between GnRH deficiency and OB atrophy in KS remains unclear.

**Added value of this study**

We identify *ZBTB20* as a CHH-associated gene through a pathogenic variant (p.R300C) that fully segregates in a large four-generation family, with further support from a significant enrichment of rare *ZBTB20* variants in our CHH cohort (n = 812). Nervous system-specific *Zbtb20* knockout mice recapitulate core CHH features, including GnRH neuron deficiency, hypogonadism, and infertility. *Zbtb20* deficiency disrupts the TN scaffold, which likely underlies the arrested migration of GnRH neurons. Moreover, *Zbtb20* loss impairs the proliferation of neural stem cells in the subventricular zone (SVZ) and disrupts neuroblast migration along the rostral migratory stream. We also identify *Thbs4* as a direct transcriptional target of ZBTB20, revealing the ZBTB20-Thbs4 axis as a key regulatory pathway in SVZ-OB neurogenesis.

**Implications of all the available evidence**

Our findings establish *ZBTB20* as a CHH-associated gene and demonstrate its roles in the SVZ-OB neurogenesis and TN development, thereby explaining the co-occurrence of OB atrophy and GnRH deficiency in patients with KS with *ZBTB20* mutations. This work expands the genetic and mechanistic understanding of CHH and provides support towards inclusion of *ZBTB20* in genetic diagnostic panels for patients with CHH.

## Introduction

Congenital hypogonadotropic hypogonadism (CHH) is a rare endocrine disorder characterised by impaired secretion or action of gonadotropin-releasing hormone (GnRH), leading to delayed or absent puberty and infertility. With an estimated prevalence of 1 in 4000–10,000 individuals, CHH arises either from defective migration of GnRH neurons from the olfactory placode to the hypothalamus during embryonic development or from postnatal defects in GnRH secretion and signalling.^1^ Clinically, CHH manifests as low levels of gonadotropins and sex steroids, with phenotypic heterogeneity ranging from isolated reproductive deficits to complex syndromic forms accompanied by anosmia (Kallmann syndrome, KS), skeletal abnormalities, or neurodevelopmental defects.^2^^,^^3^ About 60 genes have been implicated in CHH; however, the genetic aetiology remains unidentified in nearly half of CHH cases.^4^

During embryogenesis, GnRH neurons originate in the olfactory placode and migrate to the hypothalamus along specialised axonal scaffolds, including the terminal nerve (TN).^5^ Concurrently, neural stem cells (NSCs) originating in the subventricular zone (SVZ) migrate through the rostral migratory stream (RMS) to the olfactory bulb (OB).^6^ KS related genes, such as *ANOS1* and *FGFR1*, are also involved in the SVZ-RMS-OB migratory pathway.^7^^,^^8^ Postnatal defects in GnRH secretion involve dysregulation of kisspeptin signalling (KISS1/KISS1R), neurokinin B (TAC3/TACR3), or leptin pathways (LEP/LEPR), which modulate pulsatile GnRH release.^9^, ^10^, ^11^

Intriguingly, many CHH-associated genes were initially discovered through investigations of syndromic disorders with hypogonadotropic hypogonadism.^12^, ^13^, ^14^, ^15^ For instance, *CHD7* is first identified as the causative gene for CHARGE syndrome-a multisystem disorder characterised by coloboma, heart defects, choanal atresia, growth retardation, genital anomalies, and ear abnormalities.^16^ Subsequent studies revealed that *CHD7* mutations account for about 6% of CHH cases.^17^^,^^18^ *SOX10* is initially associated with Waardenburg syndrome.^19^ The observation of olfactory-bulb agenesis and hypogonadism in some *SOX10*-mutant patients led to the identification of its role in GnRH neuron ontogeny.^20^ These findings imply that certain genes involved in multisystem syndromes with hypogonadism may be associated with CHH as well.

The *ZBTB20* gene, encoding a zinc finger and BTB domain-containing protein, is a crucial transcriptional regulator involved in various physiological processes, including neurodevelopment, glucose and lipid metabolism, and immune response.^21^, ^22^, ^23^ Mutations in *ZBTB20* are linked to Primrose syndrome, a rare genetic disorder characterised by intellectual disability, skeletal abnormalities and distinctive facial features.^24^ Interestingly, some male patients with *ZBTB20* mutations are reported with gonadal hypoplasia (micropenis or cryptorchidism).^25^, ^26^, ^27^ Indeed, *Zbtb20* global knockout mice show sexual infantilism. However, its global disruption only results in a reduction in the somatotrope population and a complete loss of mature lactotropes, while other lineages of the anterior pituitary, including thyrotropes, corticotropes, and gonadotropes remain largely unaffected.^28^ Furthermore, sperm-specific *Zbtb20* ablation does not cause sterility.^29^ These studies suggest that the gonadal hypoplasia in patients with *ZBTB20* mutations likely arises through mechanisms independent of direct germ cells or pituitary defects.

In this study, we identified a loss-of-function *ZBTB20* mutation in a large CHH pedigree and four more *ZBTB20* variants in a well-recorded CHH cohort from China. Nervous-system-specific *Zbtb20* conditional knockout (cKO) mice recapitulated key CHH phenotypes, including disrupted pubertal maturation and gonadotropin deficiency. *Zbtb20* deficiency disrupts TN scaffold, which may underlie the arrested migration of GnRH neurons. Further, *Zbtb20* deficiency impaired neurogenesis in the SVZ, with cell cycle perturbations (prolonged S phase) and aberrant migration of neuronal progenitors. These findings establish *ZBTB20* as a new CHH-associated gene, warranting further validation in diverse ethnic cohorts to assess its broader pathogenic contribution.

## Methods

### Patients and clinical examinations

We recruited a four-generation KS family (L1840) from Hunan Province, China. Twenty-two members participated in this study. All subjects underwent detailed physical examinations, olfaction testing and hormone testing. In addition, brain MRI was performed on three affected members (II:2, III:1, III:7). Genomic DNA was extracted from peripheral blood samples following standard phenol-chloroform methods.

Through a 15-year, multi-centre effort to build a large Asian CHH cohort, we have established a whole-exome sequencing (WES) dataset comprising 812 patients with CHH (630 males and 182 females), with participants recruited from Peking Union Medical College Hospital (Beijing), People’s Hospital of Henan Province (Zhengzhou), Xiangya Hospital, and Hunan Children’s Hospital (Changsha). The mean age at diagnosis was 21.9 years. The patients were diagnosed with CHH according to established criteria. Diagnosis of CHH is age-dependent and requires exclusion of alternative causes. In infants (especially boys with micropenis/cryptorchidism), low gonadotropins and sex steroids during minipuberty suggest CHH. In adolescents (≥14 years in boys, ≥13 in girls), absent puberty with hypogonadotropic hypogonadism must be differentiated from constitutional delay. In adults, infertility or osteoporosis with hypogonadotropic hypogonadism warrants evaluation, excluding acquired causes like pituitary tumours. Olfactory testing and genetics aid diagnosis. For those with KS, anosmia or hyposmia confirmed through formal testing using the University of Pennsylvania Smell Identification Test (UPSIT), with scores falling below the 5th percentile for their respective age group. The control group consists of 4936 unrelated, ethnic-matched Chinese participants without CHH features.

### WES

WES was conducted in three affected (II:2, III:1, III:7) members and one unaffected (III:3) member. Genomic DNA was extracted from peripheral blood lymphocytes by means of the phenol/chloroform method. The whole exome was captured by the Agilent Sureselect Human All Exon V4+UTR enrichment platform (Agilent Technologies, Santa Clara, California) and then sequenced on the Illumina Hiseq X Ten platform (Illumina, San Diego, California) with paired-end reads of 350 bp. Generated reads were aligned to the human reference genome hg19 (UCSC version) with the use of Burrows-Wheeler aligner software (v0.7.15). Quality control was recalibrated with the use of GATK table recalibration. Duplicate reads were detected by Picard 1.14 (http://broadinstitute.github.io/picard/).

### Whole genomic sequencing (WGS)

WGS was performed on two patients (III:1 and III:7) and one unaffected (III:3) member. Copy number variation (CNV) Calls and homozygosity mapping were analysed by WGS. Integrative Genomics Viewer was used to visualise the mapping reads, read depths, and breakpoints. CNV was performed using the log2-ratio of read depth on each exon. Depth data were exported by GATK using the option for depth of coverage and whole chromosome bed files. Matrix attachment region (MAR)-Wiz was used to identify scaffold/MARs), which were related to breakage events and nonhomologous recombination. DGV database and its derived series of Stringent Lib, Inclusive Lib, and DGV Gold Standard were used to annotate the detected benign CNVs. To eliminate false positives, we assessed sequence read depth for all putative events, requiring duplication or deletion in read depth in the subjects when compared to normal parental exomes. CNVs and homozygosity mapping analysis were detected on all the CHH related and candidate genes.

### Antibodies

The antibodies used for immune-staining were as follows: mouse anti-GnRH (1:500, MAB5456-C, Millipore, RRID:AB_571014); Rabbit anti-ZBTB20 (1:500, Cat# 23987-1-AP, Proteintech, RRID:AB_2879389); Goat anti-Robo3 (1:300, AF3155, R&D systems, RRID:AB_10644167); Rat anti-PHH3 (1:400, ab10543, Abcam, RRID:AB_2295065); Rabbit anti-Ki67 (1:400, 12202s, Cell signalling, RRID:AB_2620142); Rat anti-BrdU (1:250, ab6326, Abcam, RRID:AB_305426); Alexa Fluor 488-conjugated goat anti-mouse IgG (H + L) (1:1000, ab150113, Abcam, RRID:AB_2576208); Alexa Fluor 555-conjugated goat anti-Rabbit IgG (H + L) (1:1000, ab150078, Abcam, RRID:AB_2722519); Alexa Fluor 647-conjugated goat anti-Rat IgG (H + L) (1:1000, ab150167, Abcam, RRID:AB_2864291). An anti-Flag antibody (F1804, Sigma, RRID:AB_262044) was used for ChIP assay.

### Animals

As reported previously, *Zbtb20* conventional KO mice are not viable beyond early postnatal stages due to severe growth retardation, hypoglycaemia, and premature lethality.^23^^,^^28^ To explore the pathological mechanisms underlying CHH induced by *ZBTB20* mutations, we specifically knockout *Zbtb20* gene in the central nervous system. *Zbtb20*^loxp/+^mice, with two loxP sites flanking exon 8, were generated by Cyagen Inc (Suzhou, China; Strain S-CKO-12132) by using CRISPR-Cas9 technology. *Zbtb20*^loxp/+^ mice were bred with Nestin-Cre transgenic mice (Jackson Laboratory; stock number 003771) for several cycles to obtain *Zbtb20*^loxp/loxp^: Nestin-Cre mice, which specifically knockout of *Zbtb20* in the central nervous system. Mice of the same sex were group-housed with 3–5 animals per cage under controlled conditions [temperature, 20 ± 2 °C; relative humidity, 50–60%; 12:12-h light–dark (LD) cycle, lights on at 7:00 AM and lights off at 7:00 PM] and had free access to food and water. All procedures regarding the care and use of animals were approved by the Institutional Animal Care and Use Committee of Central South University of China.

The primers for genotyping WT and *Zbtb20*^loxp/^ alleles were as follows:

*Zbtb20* forward-1: 5’- GCTTTCTTCTCAGTATGGAATGGT -3’
*Zbtb20* reverse-1: 5’- TTTACACTGTTCCCAGAAGGGAG -3’
*Zbtb20* forward-2: 5’- CATCTTCCAGATCCCAAAGAAACC -3’
*Zbtb20* reverse-2: 5’- ATTTCTACCAAGGTGACACAGTTTG -3’

The PCR product, amplified by *Zbtb20* forward-1 and *Zbtb20* reverse-1, for WT allele is 134 base pairs (bp), whereas that for *Zbtb20*^loxp/^ allele is 203 bp.

### 3D immunostaining, clearing and imaging of E14.5 embryos

The SOLID-DL protocol was applied in this study for 3D labelling of GnRH and Robo3 within the intact E14.5 embryos.^30^ In brief, fixed embryos were incubated with the primary antibody (rabbit anti-GnRH: Z26950-1-AP, 1:500, Proteintech; goat anti-Robo3: AF3155, 1:300, R&D systems) and secondary antibody (A-11037, 1:500, Thermo Fisher Scientific) for 7 days each. The embryos were then counterstained with TO-PRO-3 (T3605, 1:1000, Thermo Fisher Scientific) for 5 days to label the cell nuclei. After finishing labelling, the embryos were cleared with the SOLID clearing protocol. Cleared embryos were imaged using light-sheet microscope (Lightsheet 7, Zeiss) equipped with a 5 × objective lens (NA = 0.16).

### Calculation of cell cycle kinetic parameters

The calculation of cell cycle parameters was performed as follows: EdU (50 mg/kg, i.p.) was injected at T = 0 h to label progenitor cells in S-phase at the beginning of the experiment. BrdU (50 mg/kg, i.p.) was administered at T = 1.5 h to label cells in S-phase at the end of the labelling period (Scells). Animals were sacrificed at T = 2 h. Based on the approximately 30 min required for each label to circulate and incorporate detectably, the effective interval during which cells incorporate EdU but not BrdU (Ti) is 1.5 h. Since progenitor cells are not synchronised, a fraction of the initial EdU-labelled cohort will exit S-phase during Ti and thus be EdU^+^/BrdU^−^ (Lcells). The ratio Lcells/Scells equals Ti/Ts, allowing calculation of Ts (S-phase duration) as Ts = Ti/(Lcells/Scells). The total cell cycle length (Tc) was derived using the formula Tc = Ts/LI, where LI (labelling index) is Scells/Pcells, and Pcells represents the total number of proliferating cells in the sampling area. Additionally, the G2 duration (TG2) was determined by BrdU labelling at 0.5, 1, 1.5, and 2 h post-injection, with TG2 defined as the time when 50% of pHH3^+^ cells are BrdU^+^. The M-phase length (Tm) was calculated from the fraction of pHH3^+^ mitotic cells multiplied by Tc. For each time point and parameter, data were collected from at least 3 animals (biological replicates), and the entire experimental protocol was repeated three times to ensure reproducibility.

### Cell lines and culture

The HEK293T (ATCC® CRL-3216™) and N2a (Neuro-2a, ATCC® CCL-131™) cell lines were purchased from the American Type Culture Collection (ATCC). All cell lines were authenticated by short tandem repeat (STR) profiling and confirmed to be free of mycoplasma contamination through routine testing. Experiments were performed with cells between passages 5 and 20 post-thawing. HEK293T cells were maintained in Dulbecco’s modified Eagle’s medium (DMEM, Gibco) supplemented with 10% foetal bovine serum (FBS, Gibco), and N2a cells were cultured in Minimum Essential Medium (MEM, Gibco) containing 10% FBS. All cells were incubated at 37 °C under 5% CO_2_. Plasmid transfections were carried out using Lipofectamine 2000 Reagent (Invitrogen) in accordance with the manufacturer’s protocol.

### Immunofluorescence analysis

Tissues from 8-week-old mice were fixed in 4% paraformaldehyde overnight at 4 °C, dehydrated in graded sucrose solutions (10%, 20%, 30%), and embedded in a 1:1 mixture of OCT compound and 30% sucrose. Sections (30 μm) were sliced using a cryotome (Leica CM1850). For staining, sections were permeabilised with 0.3% Triton X-100 in PBS for 30 min, blocked in 5% BSA, 10% FBS, and 0.3% Triton X-100 in PBS for 60 min, and incubated with primary antibodies overnight at 4 °C. After PBS washes, fluorochrome-labelled secondary antibodies were applied.

To measure the nuclear localisation of ZBTB20 proteins, plasmids expressing 3 × FLAG tagged WT or mutant ZBTB20 were transfected into HEK293T cells plated on cover slides. Twenty-four hours after transfection, cells were either fixed with 4% paraformaldehyde for 10 min, or treated with CSK buffer (10 mM PIPES, pH 6.8, 100 mM NaCl, 300 mM sucrose, 3 mM MgCl2, 1 mM EGTA, 0.5% Triton X-100, with phosphatase and protease inhibitors) to remove free nuclear protein aggregates before being fixed. Cells were permeabilised with 0.3% Triton X-100 in PBS for 30 min, blocked in 5% BSA, 10% FBS, and 0.3% Triton X-100 in PBS for 60 min, and incubated with anti-FLAG antibody overnight at 4 °C. After PBS washes, fluorochrome-labelled secondary antibodies were applied. After staining, slides were mounted with DAPI and imaged using a confocal fluorescence microscope (Leica, TCS SP5).

### Measurement of hormone levels in mice

The blood taken from 8-week-old mouse retro-orbital plexuses were collected into 1.5-ml Eppendorf tubes and store at room temperature for 1 h. And serum was obtained by centrifugation for 15 min at 3000 rpm. The serum hormone levels were measured by using ELISA kits (Elabscience, Wuhan, China): FSH (E-EL-M0511), LH (E-EL-M3053), Estradiol (E-OSEL-M0008) and Testosterone (E-OSEL-M0003).

### RNA-sequencing

Total RNA was extracted from the SVZ of 8-week-old male mice using TRIzol reagent (Thermo Fisher Scientific). RNA integrity and quantity were assessed using the RNA Nano 6000 Assay Kit on a Bioanalyzer 2100 system (Agilent Technologies). The mRNA was isolated using poly-T oligonucleotide magnetic beads, followed by PCR amplification and purification with AMPure XP beads to construct the library. Library quantification was performed using a Qubit 2.0 fluorometer, and sequencing was carried out on an Illumina NovaSeq 6000. Reads were generated using CASAVA base recognition, aligned to the reference genome using Hisat2 (v2.0.5), and gene counts were obtained with featureCounts (v1.5.0-p3). Differential expression analysis was conducted using DESeq2 (v1.20.0), with adjusted P-values (Padj < 0.05) and |log2(fold change)| ≥ 1 as significance thresholds. Enrichment analysis of differentially expressed genes in KEGG pathways was performed using the clusterProfiler R package (v3.8.1), with corrected P-values < 0.05 considered significant.

### Chromatin immunoprecipitation (ChIP)

N2a cells were transfected with plasmid expressing 3 × FLAG tagged ZBTB20 or empty vector by using Lipofectamine 2000 according to the manufacturer’s protocol. Forty-eight hours after transfection, cells were crosslinked with 1% formaldehyde (10 min, room temperature), quenched with glycine, and then washed with PBS. Nuclei were isolated using sequential lysis buffers (L1: 50 mM HEPES-KOH, 140 mM NaCl, 1 mM EDTA, 0.5% NP-40, 0.25% Triton X-100; L2: 10 mM Tris-HCl, 200 mM NaCl, 1 mM EDTA; L3: 10 mM Tris-HCl, 100 mM NaCl, 1 mM EDTA, 0.5% N-lauroylsarcosine). Chromatin was sonicated (Misonix 3000), immunoprecipitated overnight with anti-FLAG antibody (Sigma, F1804), followed by incubation with Protein A/G beads (Thermo Fisher Scientific, 26162) for 2 h. After washes (50 mM HEPES-KOH, 500 mM LiCl, 1% NP-40), DNA was eluted (50 mM Tris-HCl, 1% SS), treated with RNase A/proteinase K, and purified by AMPure XP Beads (Beckman, A63881) for qPCR.

### Luciferase assay

A DNA fragment encompassing the mouse *Thbs4* promoter region (−2034 to +1 relative to the transcription start site, TSS) was cloned into the pGL3-basic vector (Promega) to generate the pThbs4-Luc reporter plasmid. N2a cells were co-transfected with the pThbs4-Luc reporter, expression plasmids for WT or mutant ZBTB20, as well as a p300 expression plasmid, using Lipofectamine 2000. Forty-eight hours after transfection, cells were lysed with Reporter Lysis Buffer (Promega). The extracts were assayed for luciferase activity with the Luciferase Assay System (Promega). Luciferase activities were determined using SIRIUS luminometer (Berthold Detection Systems GmbH).

### Statistics

Statistical analyses were performed using GraphPad Prism 8.0 (GraphPad Software, San Diego, CA, USA). Data are presented as mean ± standard error of the mean (SEM). Normality of data distribution was assessed using the Shapiro–Wilk test. Comparisons between two groups were made using an unpaired, two-tailed Student’s t-test (for parametric data) or the Mann–Whitney test (for non-parametric data). Comparisons among multiple groups were analysed by one-way analysis of variance (ANOVA) followed by Tukey’s post hoc test. A P-value of <0.05 was considered statistically significant.

Sample size determination: The sample size for animal experiments was determined a priori, using G∗Power 3.1 software based on effect sizes derived from pilot studies or previous literature, with an α error of 0.05 and a power (1-β) of 0.8. Randomisation: Animals were randomly assigned to experimental groups (WT vs. cKO) using a computer-generated random number sequence to minimise selection bias. Blinding: Investigators were blinded to the genotype (WT vs. cKO) and treatment groups during data collection (e.g., image acquisition, behavioural tests), data analysis (e.g., cell counting, hormonal assays), and outcome assessment. Inclusion/Exclusion criteria: No data points or animals were excluded from the analyses unless they met pre-established exclusion criteria, which were solely based on technical failures (e.g., unsuccessful injection, tissue processing artifacts, or poor-quality RNA/DNA) determined prior to unblinding. All efforts were made to minimise animal use and suffering.

### Ethics

Both human and animal studies were approved by the ethics committee of School of Life Science, Central South University (No.2017030801).

All participants or their legal representatives provided informed consent before joining the study. For participants under 18 years of age, written informed consent was obtained from their parents or legal guardians, and verbal/written assent was additionally obtained from the minors themselves where developmentally appropriate. All procedures were performed in accordance with the ethical standards of the responsible institutional committee and adherence with the Declaration of Helsinki principles.

Animal studies were performed in strict accordance with the ARRIVE guidelines and the NIH Guide for the Care and Use of Laboratory Animals.

### Role of funders

The funders of the study, including the National Natural Science Foundation of China and the Natural Science Foundation of Hunan Province, had no role in the study design, data collection, data analysis, data interpretation, or writing of the manuscript. All authors have full access to the data in the study and accept responsibility to submit for publication.

## Results

### Identification of a *ZBTB20* mutation in a CHH pedigree

We investigated a four-generation CHH pedigree with seven affected individuals (Fig. 1A, Supplementary Table S1). Individual I:1 presented with hyposmia and delayed puberty; II:2 showed hyposmia, right cryptorchidism, and delayed puberty, achieving conception following unspecified traditional Chinese medicine therapy; The proband (III:7) displayed KS as well as dysmorphic face, cervical scoliosis, bilateral cryptorchidism; II:6 and III:1 manifested KS with dysmorphic faces, cervical scoliosis, and mild cognitive impairment, with III:1 attaining fertility through gonadotropin therapy; IV:1, a seven years old girl, showed anosmia and mild cognitive impairment; IV:2, a five years old boy, exhibited anosmia, dysmorphic face, mild cognitive impairment, bilateral cryptorchidism and micropenis, highly suggestive of hypogonadism.

**Fig. 1:**
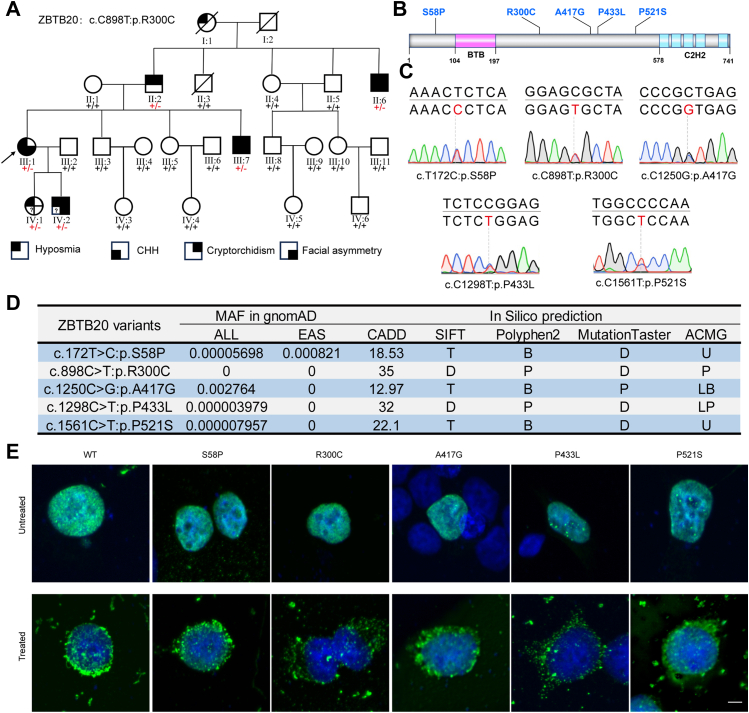
Identification and pathogenicity assessment of *ZBTB20* variants in patients with CHH. (A) Family pedigree carrying *ZBTB20 R300C*. Squares denote males and circles denote females. (B) ZBTB20 domain structure (purple box, BTB domain; Light blue frame, zinc finger) and the location of the variants found in patients with CHH. (C) Sequencing results from patients with *ZBTB20* variants. (D) *ZBTB20* variants were assessed for their potential pathogenicity using a combination of population genetics and in silico prediction tools. Allele frequency was obtained from the gnomAD (public release v2.1.1, GRCh37/hg19) database (EAS: East Asian population; ALL: all populations). ACMG criteria: P, pathogenic; B, benign; U, uncertain significance; LP, likely pathogenic; LB, likely benign. The p.R300C (PS3+PS4+PM1+PM2+PP1+PP3+PP4) as P and p.P433L (PS4+PM2+PP2+PP4) as LP according to ACMG. CADD: the scores ≥20 suggests a potentially deleterious variant, ≥30 indicates high confidence for damaging effects, and scores below 10–15 are typically considered benign. SIFT: T, tolerated; D, deleterious. PolyPhen-2: B, benign; P, possibly damaging; D, damaging. Mutation Taster: N, polymorphism; D, disease causing; A, disease_causing_automatic; P, polymorphism automatic. (E) Confocal microscopy analysis of cells expressing wild-type ZBTB20 and five variants (green), either without treatment (upper) or treated with CSK buffer (lower) before fixation. CHH, congenital hypogonadotropic hypogonadism; ACMG, American College of Medical Genetics and Genomics. Scale bar, 5 μm.

WES was conducted in three affected members (II:2, III:1, III:7) and one unaffected member (III:3). The average sequencing depth was 50 × and minimal coverage for targeted region by 10 × was 97.9%. We first screened rare sequencing variants (RSVs, MAF ≤ 0.01) in a virtual CHH gene panel with known and candidate CHH genes (Supplementary Table S2). As a result, a total of 75 RSVs were identified in the exomes of three affected members but not in the exome of control. We then performed co-segregation analysis on the 75 RSVs by using PCR-Sanger sequencing. As a result, only *ZBTB20* c.898C > T (p.R300C) co-segregated with CHH in this pedigree. This mutation was not reported in East Asian population (EAS) in Genome Aggregation Database (gnomAD, public release v2.1.1, GRCh37/hg19). It did not present in a 4936 ethnically matched WES database recruited by Xiangya hospital. *ZBTB20* R300C was predicted to be deleterious by SIFT, PolyPhen-2, Mutation Taster, CADD and American College of Medical Genetics and Genomics (ACMG). This arginine residue showed evolutionary conservation across vertebrates (Supplementary Fig. S1). No CNV in known or candidate CHH genes was found to co-segregate with CHH after WGS in two affected patients (III:1, III:7) members and one unaffected (III:3) member.

### *ZBTB20* mutations identified in sporadic patients with CHH

We further screened *ZBTB20* RSVs in the WES date from additional 812 CHH probands. As a result, four more *ZBTB20* RSVs were identified in seven patients (Fig. 1B–D, Supplementary Table S3). The variant *ZBTB20* p.S58P (rs1454955241) was present in three sporadic KS cases. In spite of one patient carrying a compound heterozygous PNPLA6 mutation, the other two patients have no mutation in known CHH-related genes. However, this variant has a MAF of 0.0008 in the EAS in gnomAD, and it was also seen in our ethnically matched WES data. As it was predicted to be benign or tolerable by several tools, it is thus considered as a polymorphism.

The *ZBTB20* p.A417G (rs897755609) was identified in a sporadic case (L1759), who also carried the FGFR1 p.Q491X mutation and the PROKR2 p.W178S mutation. The *ZBTB20* p.A417G was also considered as a polymorphism as it was predicted to be benign or tolerable by several tools. The variant *ZBTB20* P433L (rs752208031), with a MAF of 0.000007 in the EAS in gnomAD, was identified in one male KS without other mutations in known CHH genes. *ZBTB20* P521S (rs1333489573), with a MAF of 0.000007 in all populations and not reported in the EAS in gnomAD, was identified in one patient with KS without mutations in known CHH genes. Both *ZBTB20* P433L and P521S were predicted to be deleterious or possibly damaging by at least two in silico prediction software (Supplementary Table S3).

Using the matched control group of 4936 individuals with similar ethnic, geographic, and age characteristics, we applied the same screening criteria to identify rare *ZBTB20* variants. Burden test analysis comparing the prevalence of rare *ZBTB20* variants revealed a significant enrichment in patients with CHH (CHH group: 0.86%, 7/812; control group: 0.14%, 7/4936; OR = 6.12, P < 0.001, unpaired t-test).

### The R300C and P433L mutations impaired the chromatin-affinity of ZBTB20

As a transcription factor, ZBTB20’s chromatin-binding properties were investigated to assess the impact of CHH-associated mutations. WT and mutant *ZBTB20* plasmids were transiently transfected into HEK293T cells and the subcellular localisation of ZBTB20 proteins was visualised with confocal immunofluorescence microscopy (Supplementary Table S4). Using CSK buffer to selectively remove unbound nuclear protein aggregates, we observed distinct differences in chromatin interaction between WT and mutant proteins. Both WT and mutant ZBTB20 proteins initially localised within the nuclei (Fig. 1E). However, following CSK buffer treatment, the R300C and P433L mutants exhibited cytoplasmic diffusion, while WT and other mutant forms maintained nuclear localisation. This differential response demonstrates that the R300C and P433L mutations reduced ZBTB20’s chromatin affinity.

### *Zbtb20*-deficient mice recapitulated CHH phenotypes

To investigate the mechanisms underlying the reproductive deficits in patients with *ZBTB20* mutations, we generated nervous system-specific *Zbtb20* cKO mice (Supplementary Fig. S2A and B). These mice survived to adulthood without observable viability defects, in contrast to the perinatal lethality reported for conventional global knockouts.^28^ Immunofluorescence analysis confirmed the absence of ZBTB20 protein throughout the brain of cKO animals (Supplementary Fig. S2C), thereby enabling subsequent phenotypic characterisation.

*Zbtb20 cKO* mice showed the following CHH-related phenotypes: (1) Hypogonadism. Both male and female *Zbtb20* cKO mice showed marked reductions in gonadal size and weight compared to WT controls (Fig. 2A and B). Specifically, testis weight in males and uterine size in females were significantly decreased (Fig. 2C–F). The gonadal weight/body weight ratio was also significantly lower in *Zbtb20* cKO mice than in WT controls (Fig. 2D, G). (2) Hypogonadotropic hypogonadism. Serum levels of FSH and LH were significantly reduced in both male and female *Zbtb20* cKO mice (Fig. 2H and I). (3) Infertility. Both male and female *Zbtb20* cKO mice were completely sterile, failing to produce offspring during 5 cycles mating trial (Fig. 2J). (4) GnRH neuron deficiency. The adult *Zbtb20* cKO mice showed a pronounced reduction of GnRH neurons in the medial preoptic area (MPOA) and the median eminence (ME) (Fig. 2K–M). These findings collectively demonstrate that *Zbtb20* deletion recapitulates key features of CHH, including GnRH deficiency, hypogonadism, and infertility.

**Fig. 2:**
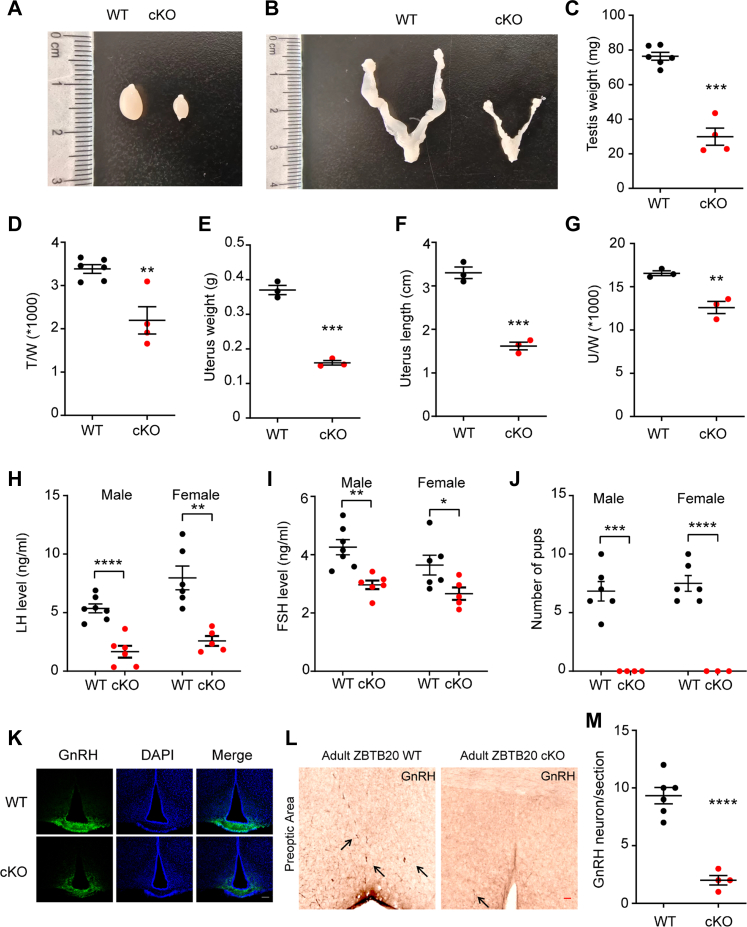
*Zbtb20* knockout caused hypogonadism and reduced number GnRH neurons in mice. (A) Representative testes from WT and cKO males at 8 weeks. (B) Representative uteri from WT and cKO females at 8 weeks. (C) Testis weight (n = 6 WT, n = 4 cKO). (D) Testis-to-body weight ratio (n = 6 WT, n = 4 cKO). (E) Uterus weight (n = 3 WT, n = 3 cKO). (F) Uterus length (n = 3 WT, n = 3 cKO). (G) Uterus-to-body weight ratio (n = 3 WT, n = 3 cKO). (H) Serum LH levels in males (n = 7 WT, n = 6 cKO) and females (n = 6 WT, n = 5 cKO). (I) Serum FSH levels in males (n = 7 WT, n = 6 cKO) and females (n = 6 WT, n = 5 cKO). (J) Fertility assessment: WT or cKO mice paired with fertile WT mates (males: n = 6 WT, n = 4 cKO; females: n = 6 WT, n = 3 cKO). VNO, vomeronasal organ; OE, olfactory epithelium; POA, preoptic area; ME, median eminence. Data are presented as means ± SEM, ∗P < 0.05, ∗∗P < 0.01, ∗∗∗P < 0.001, ∗∗∗∗P < 0.0001, unpaired t-test. (K) Immunofluorescence of GnRH in the median eminence (ME) at 8 weeks (n = 6 WT, n = 4 cKO). Scale bar: 100 μm. (L) GnRH neuron distribution in the POA of adult mice. Fewer GnRH^+^ neurons (arrows) are present in cKO mice. (M) Quantification of GnRH^+^ neurons (right; n = 6 WT, n = 4 cKO). Scale bar: 50 μm.

### The impaired migration of GnRH neurons in *Zbtb20*-deficient embryos

To visualise the three-dimensional (3D) migratory trajectories of GnRH neurons, we performed whole-mount immunofluorescence on intact E14.5 embryos using advanced tissue clearing and light-sheet imaging techniques. In WT embryos, a continuous stream of GnRH neurons migrated from the olfactory epithelium toward the hypothalamus (Fig. 3A and B). In contrast, GnRH neuron migration was abnormally stalled in the nasal-forebrain area in *Zbtb20*-deficient embryos (Fig. 3C–H, Supplementary Videos S1 and S2), magnification images in different key regions showed that there was no GnRH neuron in the hypothalamus of *Zbtb20*-deficient embryos (Fig. 3E–G). No genotypic difference in GnRH neurons was found in the nose (Fig. 3E, H). Taken together, our data indicated that *Zbtb20* deficiency impaired GnRH neuron migration.

**Fig. 3:**
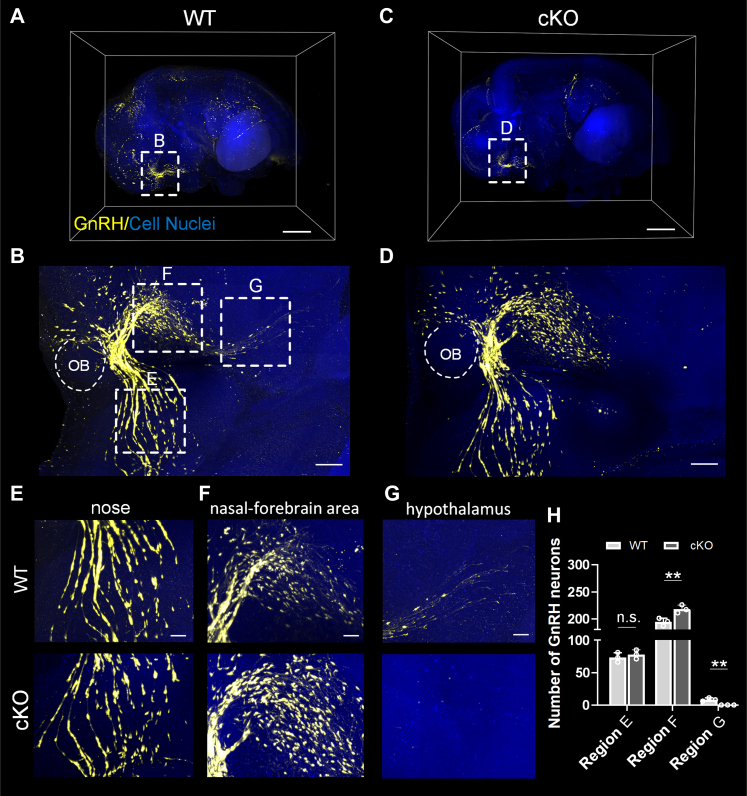
*Zbtb20* knockout impaired GnRH neuron migration. (A) 3D visualisation of GnRH neuron migration of WT mice at 14.5 days of intact embryo (E14.5). Scale bar, 1 mm. (B) Magnification of the entire migration path of GnRH neurons marked in A. Scale bar, 200 μm. (C) 3D visualisation of GnRH neuron migration of *Zbtb20* cKO embryos at E14.5. Scale bar, 1 mm (D) Magnification of the entire migration path of GnRH neurons marked in C. Scale bar, 200 μm. (E–G) Magnifications of key regions along the migration path marked in B, nose for E, nasal-forebrain area for f and hypothalamus for G, respectively. Scale bar, 100 μm. (H) Quantitative analysis of GnRH neuron distribution in key regions in WT and cKO embryos (n = 3/genotype). Data are presented as means ± SEM, ∗∗P < 0.01, unpaired t-test.

### Olfactory bulb hypoplasia in *Zbtb20* cKO mice

In addition to CHH phenotypes, *Zbtb20* cKO mice exhibited significant reductions in body size and weight compared to WT littermates (Fig. 4A, Supplementary Fig. S3). Postnatal brain weight was also markedly decreased in *Zbtb20* cKO mice (Fig. 4B, E). As some patients with *ZBTB20* mutations present with anosmia/hyposmia, we also compared the OB in WT and *Zbtb20* cKO mice. Notably, the OB of *Zbtb20* cKO mice showed pronounced volumetric atrophy, with both absolute weight and OB-to-brain weight ratio being significantly reduced relative to WT controls (Fig. 4F and G). Histological analysis of coronal and sagittal sections through H&E staining confirmed structural hypoplasia of the OB in *Zbtb20* cKO mice (Fig. 4C and D).

**Fig. 4:**
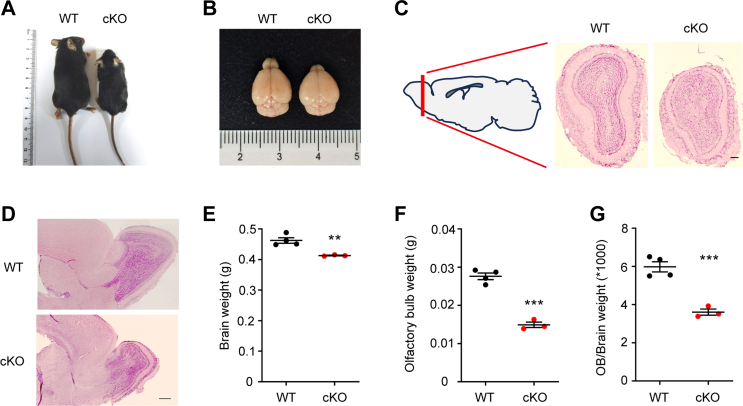
*Zbtb20* knockout impaired OB development. (A) Gross morphology of 8-week-old WT and *Zbtb20* cKO mice. (B) Whole-brain view of WT and *Zbtb20* cKO mice at 8 weeks. (C) Haematoxylin and eosin (H&E) staining of the OB at 8 weeks. Scale bar: 200 μm. (D) H&E staining of the RMS. Scale bar: 500 μm. (E) Quantification of the brain weight of *Zbtb20* mice at 8 weeks (n = 4 for WT and n = 3 for cKO). (F) Quantification of the OB weight of *Zbtb2*0 WT and cKO mice at 8 weeks (n = 4 for WT and n = 3 for cKO). (G) Quantitative determination of OB weight to brain weight ratio in *Zbtb2*0 WT and cKO mice at 8 weeks of age (n = 4 for WT and n = 3 for cKO). RMS, rostral migratory stream. Data are presented as means ± SEM, ∗∗P < 0.01, ∗∗∗P < 0.001, unpaired t-test.

### *Zbtb20* deficiency prolongs S phase in neural stem cell proliferation

The NSCs within the SVZ are continually differentiated and migrated to the OB in the embryonic and adult brain via the RMS.^31^ To investigate the cellular basis of OB atrophy, we analysed single-cell RNA sequencing (scRNA-seq) data from the SVZ reported by Silla et al.^32^ *Zbtb20* showed predominant expression in the NSCs, and B cells which have properties of specialised support cells known as astrocytes, implicating its functional relevance in NSCs dynamics (Supplementary Fig. S4).

EdU pulse-labelling (50 mg/kg, i.p.) revealed significantly fewer proliferating (EdU+) NSCs in adult *Zbtb20* cKO mice vs. WT littermates at 2 h post-injection (Fig. 5A and B). To delineate cell cycle perturbations, we performed BrdU/EdU dual-pulse labelling with a 1.5 h interval (Fig. 5C). Quantitative analysis demonstrated prolonged total cell cycle duration in cKO NSCs (cKO 16.0 ± 0.3 h vs. WT 14.5 ± 0.2 h; P < 0.05, unpaired t-test), attributable to specific extension of S phase (cKO 11.0 ± 0.4 h vs. WT 9.0 ± 0.3 h; P < 0.05, unpaired t-test) (Fig. 5D, F). Consequently, the S phase fraction was significantly elevated in mutants (Fig. 5G and H). In contrast, G2/M phase durations remained unchanged between genotypes: G2 phase (PHH3+/BrdU+) persisted for ∼1 h and M phase (PHH3+/Ki67+) for ∼0.8 h in both groups (Fig. 5E, I–K).

**Fig. 5:**
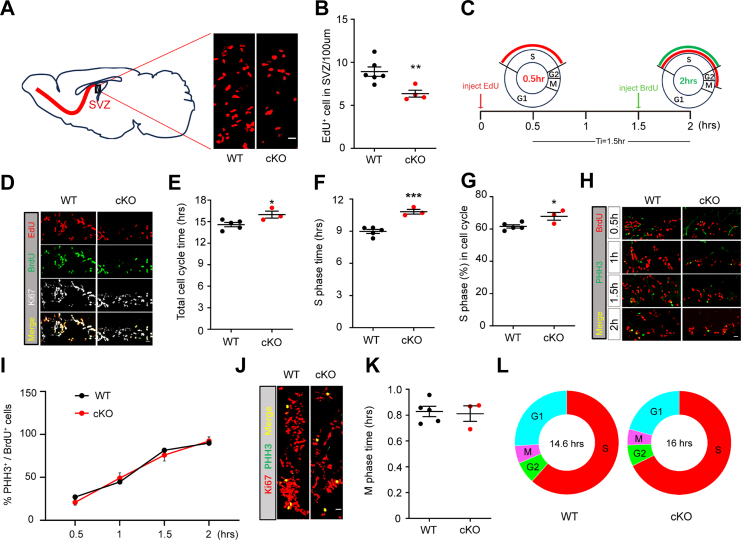
*Zbtb20* deletion altered neural stem cell proliferation and cell cycle dynamics in the SVZ. (A) Immunofluorescence imaging of EdU stained by SVZ after EdU injection for 2 h in *Zbtb2*0 WT and cKO mice at 8 weeks of age. Scale bar, 20 μm. (B) Quantification of EdU^+^ cells per 100 μm SVZ (n = 6 WT; n = 4 cKO). (C) To analyse cell cycle kinetics, pregnant mice were injected with EdU at T = 0 h to label all S-phase cells. At T = 1.5 h, BrdU was administered to mark cells still in S-phase at the end of the labelling window, followed by fixation 0.5 h later. Cells that incorporated only EdU (EdU^+^ BrdU^−^), designated as the leaving fraction (Lcells), represent those that exited S-phase during the 1.5 h interval between EdU and BrdU injections (Ti). (D) Immunofluorescence for EdU (red, initial S-phase), BrdU (green, late S-phase), and Ki67 (grey, proliferation marker). Scale bar: 20 μm. (E) Total cell cycle duration (n = 5 WT; n = 3 cKO). (F) S-phase length (n = 5 WT; n = 3 cKO). (G) Fraction of cell cycle spent in S-phase (n = 5 WT; n = 3 cKO). (H) G2/M progression assay: BrdU (red) and PHH3 (green, mitotic marker) staining at 0.5–2 h post-BrdU. Scale bar: 20 μm. (I) G2-phase duration (n = 5 WT; n = 3 cKO). (J) Co-staining of Ki67 (red, cycling cells) and PHH3 (green, mitosis). Scale bar: 20 μm. (K) M-phase length (n = 5 WT; n = 3 cKO). (L) Pie chart of SVZ cell cycle. SVZ, subventricular zone. Data are presented as means ± SEM, ∗P < 0.05, ∗∗P < 0.01, ∗∗∗P < 0.001, unpaired t-test.

### *Zbtb20* deficiency impairs NSCs migration

To assess potential migration defects, we performed EdU pulse-chase labelling (50 mg/kg, i.p.) in adult mice and analysed cell distribution 5 days post-injection. Sagittal section immunofluorescence revealed significantly fewer EdU + cells along the entire migratory route from the SVZ through the RMS to the OB in *Zbtb20* cKO mice compared to WT controls (Fig. 6A–C, Supplementary Fig. S5).

**Fig. 6:**
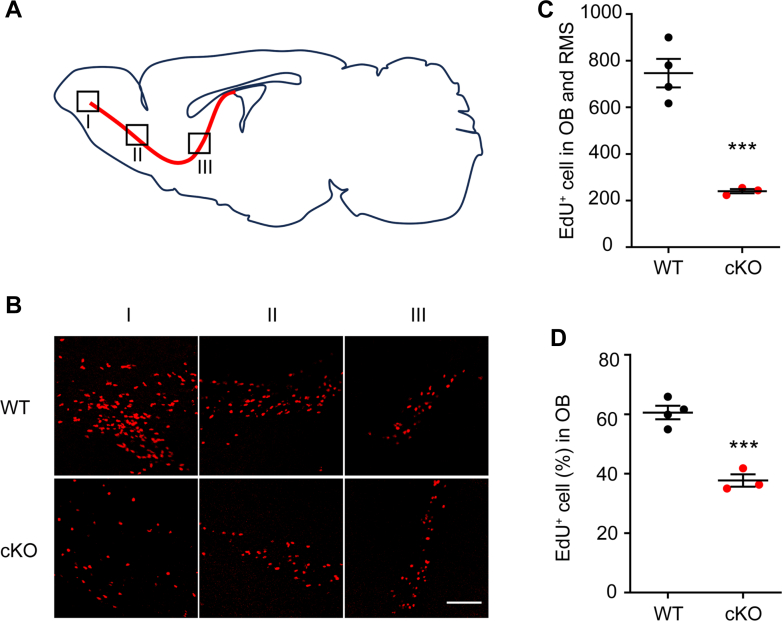
Stem cell migration in the SVZ was affected in *Zbtb20* cKO mice. (A) The RMS of mice was mapped, and three regions were selected for statistical purposes (B) Immunofluorescence imaging of EdU stained by RMS after EdU injection for 5 days in *Zbtb2*0 WT and cKO mice at 8 weeks of age. Scale bar, 100 μm. (C) Quantification of cells expressing EdU (red) in OB and RMS after EdU injection for 5 days in *Zbtb2*0 WT and cKO mice at 8 weeks (n = 4 WT; n = 3 cKO). (D) Quantitative determination of the proportion of EdU positive cells in the OB in *Zbtb2*0 WT and cKO mice at 8 weeks (n = 4 WT; n = 3 cKO). Data are presented as means ± SEM, ∗∗∗P < 0.001, unpaired t-test.

Quantitative analysis demonstrated altered migratory dynamics: while the proportion of EdU + cells retained in the SVZ and RMS was significantly increased in mutants, fewer cells successfully reached the OB (Fig. 6D). These findings indicate that *Zbtb20* is required for efficient NSCs migration along the RMS-OB pathway.

### ZBTB20 transcriptionally regulates the expression of *Thbs4*

To investigate the molecular mechanisms underlying the *Zbtb20* cKO phenotype, we performed RNA sequencing of the SVZ from *Zbtb20* cKO and WT mice. This analysis revealed 159 upregulated and 237 downregulated genes in cKO mice (Fig. 7A). KEGG pathway enrichment showed significant alterations in the extracellular matrix (ECM) receptor interactions and Wnt/β-catenin signalling pathways (Fig. 7B, Supplementary Fig. S6).

**Fig. 7:**
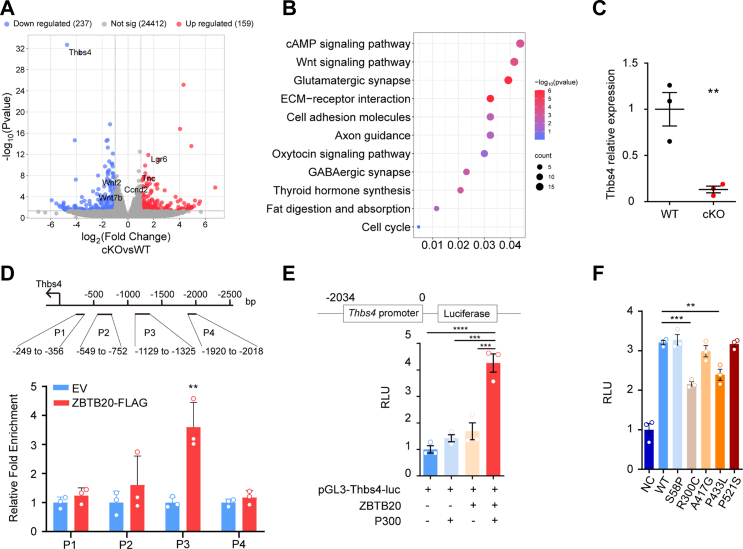
ZBTB20 up-regulated Thbs4 gene transcription. (A) Volcano map of SVZ differentially expressed genes in *Zbtb2*0 WT and cKO mice at 8 weeks of age. (B) KEGG pathway analysis of differentially expressed genes. (C) The mRNA levels of Thbs4 in 8-week-old *Zbtb20* cKO mice were down-regulated by real-time quantitative PCR compared with WT. (D) Following transfection of wild-type ZBTB20 or empty vector plasmids into N2a cells, chromatin immunoprecipitation (ChIP) was performed using a Flag antibody. The precipitated DNA was amplified with four distinct primer pairs targeting the Thbs4 promoter region to determine its relative fold enrichment. (E) Following transfection of N2a cells with either empty vector, P300-myc plasmid, wild-type ZBTB20 plasmid, or a combination of P300-myc + wild-type ZBTB20 plasmids (co-transfected with the Thbs4-luciferase reporter plasmid), cell lysates were prepared to measure luciferase activity. (F) Following transfection of N2a cells with wild-type ZBTB20 plasmid or mutant plasmid (co-transfected with P300-myc plasmid), cell lysates were prepared to measure luciferase activity. Data are presented as means ± SEM, ∗∗P < 0.01, ∗∗∗P < 0.001, unpaired t-test.

Quantitative real-time PCR (qRT-PCR) confirmed that *Thbs4* mRNA levels were significantly downregulated in 8-week-old *Zbtb20* cKO mice (Fig. 7C, Supplementary Table S5). Further, ChIP assays were performed in N2a cells expressing FLAG-tagged ZBTB20 using an anti-Flag antibody (Supplementary Table S6). Analysis of precipitated DNA by qPCR with primers spanning four regions of the *Thbs4* promoter revealed significant enrichment at the region amplified by primer pair 3 (−1129 to −1325 bp), indicating direct binding of ZBTB20 to the *Thbs4* promoter (Fig. 7D).

To further validate ZBTB20-mediated transcriptional regulation, a luciferase reporter assay containing the mouse *Thbs4* promoter was employed. Co-expression of ZBTB20 with the transcriptional coactivator P300 led to a significant increase in luciferase activity, whereas neither ZBTB20 nor P300 alone induced activation (Fig. 7E). Consistently, ZBTB20 and P300 synergically increased the mRNA level of *Thbs4* in N2a cells (Supplementary Fig. S7). These findings demonstrate that ZBTB20 directly activates *Thbs4* transcription via promoter binding, requiring P300 as a coactivator.

We also evaluated the functional impact of CHH-associated *ZBTB20* mutations on transcriptional regulation through the luciferase reporter assay. Consistent with chromatin affinity data, luciferase activity in cells expressing *ZBTB20* variants S58P, A417G, or P521S was comparable to WT, whereas R300C and P433L mutants showed significantly reduced activity (Fig. 7F).

### Defects of terminal nerve (TN) scaffolds in *Zbtb20*-deficient embryos

Previous studies have established that the TN plays a pivotal role in guiding GnRH neuron migration by providing an essential scaffold along their migratory pathway.^5^^,^^33^ To investigate whether TN was affected by *Zbtb20* deficiency, we first re-analysed the scRNA-seq data from whole embryonic mouse noses as reported by Amato et al.^5^ As shown in Supplementary Fig. S8A and B, *Zbtb20* was expressed in the TN/pioneer cell population in the nasal region and co-expressed with TN markers. The expression of Zbtb20 in the TN neurons was further validated by double immunofluorescence staining, as Zbtb20 colocalised with Robo3, a typical TN marker,^33^ in the nose (Supplementary Fig. S8C). In contrast, Zbtb20 did not co-localise with GnRH (Supplementary Fig. S8D).

We then used Robo3 antibody to trace the TN neurons in the E14.5 embryos. As shown in Fig. 8A, Robo3-positive TN neurons distributed from the nose to the hypothalamus in a 3-D manner. Therefore, a combination of several plane is required to overview the pattern. Interestingly, Robo3 and GnRH neurons are closely interwoven in the path as demonstrated by both 3-D and 2-D immunofluorescence staining (Fig. 8A and B; Supplementary Fig. S9). However, neither Robo3 nor GnRH neuron was found in the hypothalamus region of *Zbtb20*-deficient embryos (Fig. 8C and D).

**Fig. 8:**
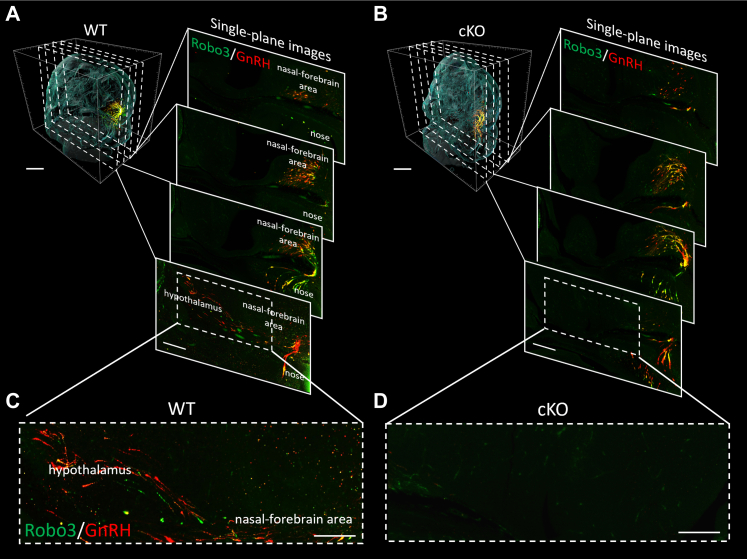
*Zbtb20*-deficiency disrupted the TN development. (A) 3D rendering and representative 2D slices of Robo3/GnRH distribution in a WT embryo at E14.5. Scale bar, 1 mm. (B) 3D rendering and representative 2D slices of Robo3/GnRH distribution in a *Zbtb20* cKO embryo at E14.5. Scale bar, 1 mm for 3D and 200 μm for 2D. (C) Magnified view of single-plane images of Robo3/GnRH distribution marked in A. Scalebar, 100 μm. (D) Magnified view of single-plane images of Robo3/GnRH distribution marked in B. Scalebar, 100 μm.

## Discussion

In this study, we identified *ZBTB20* as a genetic determinant of CHH by integrating data from human genetics and cKO mouse models. WES and WGS revealed a heterozygous ZBTB20 p.R300C variant that co-segregated with hypogonadism in a CHH pedigree. Targeted sequencing of a Chinese CHH cohort identified four additional heterozygous *ZBTB20* RSVs, with a significant enrichment of *ZBTB20* RSVs in CHH cases compared with controls. In mice, *Zbtb20* deficiency impaired SVZ neural stem cell proliferation, disrupted RMS-dependent neuroblast migration to the OB, and altered TN structure as well as GnRH neuron migration. Molecular characterisation revealed that ZBTB20 directly activates *Thbs4* transcription. Together, these findings establish ZBTB20 as a critical regulator of both SVZ-OB neurogenesis and GnRH neuron development.

Many CHH-associated genes regulate neuronal migration, axon guidance, or OB development.^4^ For instance, *ANOS1* encodes anosmin-1, an ECM protein critical for OB development and axonal guidance, and mutations in *ANOS1* cause X-linked KS.^11^ Anosmin-1 induces neurite outgrowth and cytoskeletal rearrangements through enhancing FGFR1 signalling in human embryonic GnRH olfactory neuroblasts.^34^ The FGF8/FGFR1 signalling pathway is essential for OB development and GnRH neuron migration. Disruption of this pathway, which accounts for approximately 10% of CHH cases, impairs olfactory axon guidance and compromises the migratory corridor for GnRH neurons.^35^, ^36^, ^37^ Prokineticin signalling through PROKR2 and its ligand PROK2 is essential for OB morphogenesis and GnRH neuron development. Impaired PROKR2 signalling disrupts the organisation of the olfactory nerve pathway and compromises GnRH neuron migration, resulting in CHH accompanied by variable olfactory deficits.^5^^,^^38^^,^^39^ However, it should be noted that although OB malformations frequently co-occur with CHH, evidence suggests this association may be correlative rather than causative. For instance, loss of *Arx1* does not prevent GnRH neurons from migrating to the hypothalamus, even though *Arx1*-deficient mice develop severe olfactory bulb aplasia/hypoplasia.^39^ Furthermore, olfactory defects in some families with KS do not always co-segregate with CHH.^40^

In this study, we demonstrated that *Zbtb20* deficiency impairs SVZ-OB development and GnRH neuron migration, which may underlie the aetiology of anosmia/hyposmia and CHH associated with *ZBTB20* mutations. Although the observed OB hypoplasia in *Zbtb20* cKO mice could potentially contribute to infertility through impaired olfaction or pheromone sensing, the pronounced reduction in GnRH neurons (Fig. 3) and low circulating levels of LH and FSH in these animals indicate that failure of the hypothalamic-pituitary-gonadal (HPG) axis constitutes the primary mechanism underlying reproductive deficiency.

Additionally, we revealed that *Zbtb20* deficiency impairs TN integrity, which may be the leading cause of abnormal GnRH neuron migration. The TN is thought to serve as a migratory scaffold for GnRH neurons.^33^ Re-analysis of published scRNA sequencing data indicated that Zbtb20 is expressed in TN neurons, supporting the possibility that *Zbtb20* may play a cell-autonomous role in TN neuron development. Indeed, our 3D reconstruction results further identified defects in TN development in *Zbtb20* cKO embryos (Fig. 8), which likely underlie the arrested migration of GnRH neurons. Interestingly, the migration of GnRH neurons was arrested right behind the disrupted TN scaffold.

*Prokr2*, identified as a downstream target gene of ZBTB20, plays a pivotal role in regulating circadian rhythms. ZBTB20 directly binds to the *Prokr2* promoter, enhancing its expression in the suprachiasmatic nucleus, the central circadian pacemaker.^41^ However, we did not observe significant changes in *Prokr2* expression in the SVZ of *Zbtb20* cKO mice, implying alterative mechanisms for *ZBTB20* mutations in the aetiology of CHH. These findings highlight the context-dependent regulation between ZBTB20 and its targets. Given that *Prokr2* is also highly expressed in the TN,^41^ it would be worthwhile to investigate whether ZBTB20 regulates *Prokr2* expression in this context.

Our demonstration that *Zbtb20* depletion impairs NSCs proliferation by prolonging S-phase duration underscores the highly context-dependent nature of Zbtb20’s roles across various cellular processes, including differentiation and apoptosis.^21^^,^^42^ For instance, in liver regeneration, ZBTB20 promotes cellular proliferation through induction of cyclin D1 and modulation of AKT activation.^43^ ZBTB20 overexpression promoted cell proliferation, while its knockdown using small interfering RNA suppressed proliferative capacity in non-small cell lung cancer cell lines.^44^ Similarly, during neocortical development, it facilitates astrocytogenesis via functional collaboration with Sox9 and NFIA.

Our transcriptomic analysis revealed significant dysregulation of two key pathways Wnt/β-catenin signalling and ECM pathway (Supplementary Fig. S6). The Wnt/β-catenin pathway, a critical regulator of development and tissue homoeostasis, promotes cell proliferation and differentiation by facilitating β-catenin nuclear translocation and activation of targets such as c-Myc and Cyclin D1.^45^^,^^46^ Although ZBTB20 has been shown to modulate this pathway in hepatocellular carcinoma through PPARγ repression,^47^ the precise mechanistic link between *Zbtb20* depletion and Wnt pathway dysregulation in the SVZ niche remains unclear and warrants further study.

Similarly, ECM-related pathways were markedly altered, with the most significant downregulation of *Thbs4*, a secreted glycoprotein implicated in cell adhesion, migration and tissue remodelling.^48^^,^^49^ Molecular studies demonstrated that ZBTB20 promoted *Thbs4* transcription by binding directly to *Tbhs4* promoter (Fig. 6). *Thbs4* is specifically and highly expressed in the SVZ and along the RMS. Interestingly, both *Zbtb20* and *Thbs4* were expressed in the GFAP-positive astrocytes (Supplementary Fig. S4b).^21^^,^^48^ *Thbs4* knockout resulted in impaired migration of newly formed neurons along the RMS, characterised by reduced neuronal egress from the RMS and diminished recruitment of newborn neuronal precursors to the OB. Furthermore, *Thbs4*-deficient mice exhibited severe deficits in cortical injury-induced astrogenesis within the SVZ.^48^ Collectively, our data suggest that *Thbs4* potentially contributes to the NSCs migration deficits observed in *Zbtb20* cKO mice.

Notably, Primrose syndrome arises predominantly from heterozygous *de novo* missense variants clustered in the C-terminal zinc finger domains of *ZBTB20*, which disrupt residues critical for DNA binding.^24^ In contrast, none of the *ZBTB20* variants identified in our study is localised in the five C2H2 zinc fingers. Such genotype-phenotype disparities are well-documented in CHH.^50^^,^^51^ For example, pathogenic variants in *SOX10* are associated with both Waardenburg syndrome and CHH. However, *SOX10* loss-of-function (LoF) mutations (e.g., frameshifting indels, nonsense, and splice-altering variants) are enriched in Waardenburg syndrome (79% LoF vs. 21% missense), whereas missense variants dominate in CHH (70% missense vs. 30% LoF).^50^ Similarly, most patients with CHH carrying truncating *CHD7* variants exhibit multiple CHARGE syndrome features, whereas those with missense variants typically present with CHH.^51^ These observations collectively suggest that the HPG axis may represent the most vulnerable system in syndromes with overlapping genetic aetiologies.

Although our study establishes *ZBTB20* as a candidate gene of CHH and elucidates its role in SVZ-OB neurogenesis and GnRH neuron migration, several limitations should be considered. First, the functional validation of *ZBTB20* variants was primarily based on *in vitro* assays and a cKO mouse model, which may not fully recapitulate pathophysiology in patients with CHH. And the interpretation of mutation impact is further complicated in cases where patients carry multiple variants. The relative weight of each variant, as well as potential epistatic interactions between them, remains to be solved. Second, the CHH cohort is predominantly of Chinese Han ancestry, which may limit the generalisability of our findings to other ethnic populations. Third, the incomplete penetrance and variable expressivity of *ZBTB20* mutations suggest the potential influence of genetic modifiers or environmental factors, which were not explored in this study. Future work involving multi-ethnic cohorts, functional studies in human-derived cellular models, and investigation of modifier genes will be essential to fully understand the clinical spectrum and mechanistic basis of *ZBTB20*-related CHH.

## Contributors

J.G, conducting main experiments; J.Z, conducting advanced tissue-clearing and whole-brain imaging techniques. Y.L, M.N, and R.Z, acquiring data; J.Z, Z.C, H.L and D.C analysing data; M.M designing research studies and writing the manuscript; J-D. L designing research studies, providing reagents and revise the manuscript.

J-D. L and M.M had full access to all the data in the study and verified the underlying data. All authors reviewed, revised, and approved the final manuscript.

## Data sharing statement

The RNA-seq and WES data generated in this study have been deposited in public repositories under accession numbers (GSE308208). Human genetic data are available under managed access to protect participant privacy. Requests can be directed to corresponding author (lijiada@sklmg.edu.cn) and will be processed within four weeks. All other data supporting the findings are available within the article and its Supplementary materials.

## Declaration of interests

The authors declare that they have no conflict of interest.
