# Supplementary material for: Establishing the role of ZBTB20 mutations in GnRH deficiency and impaired neurogenesis in the subventricular zone: a human cohort and animal model study
Source: eBioMedicine. 2026 Aug 4;131:106416. doi: 10.1016/j.ebiom.2026.106416 (PMC13470097; doi:10.1016/j.ebiom.2026.106416)
Supplement: Supplementary file 1 [file mmc1.pdf]

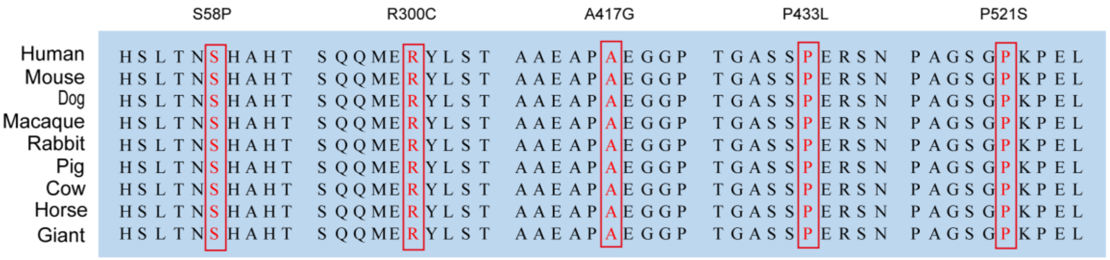

Figure S1. The residues highlighted in red represent *ZBTB20* mutation sites across species (S58P, R300C, A417G, P433L, P521S). The corresponding coding regions of these mutation sites are highly conserved among different species.

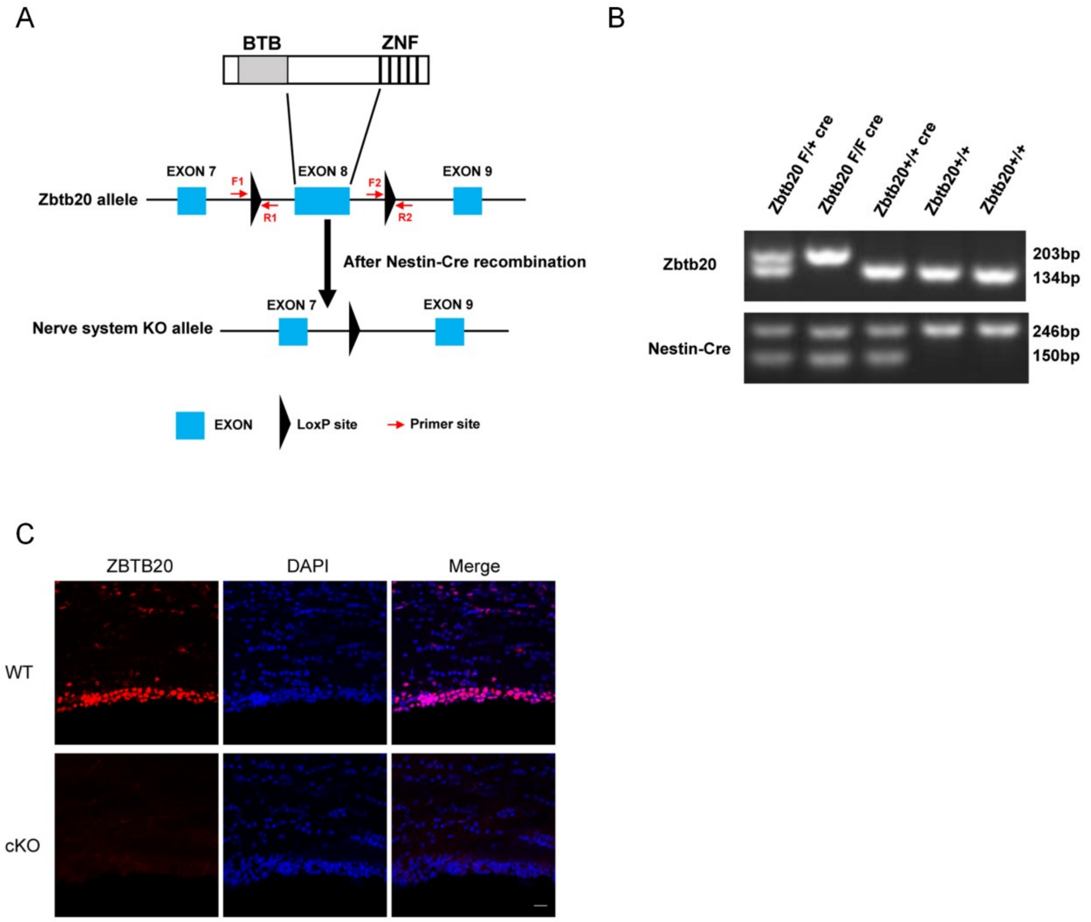

Figure S2. (A) Construction strategy of *Zbtb20* conditioned mice, knockout of exon 8. (B) PCR verification of knockout results. (C) SVZ immunofluorescence images of *Zbtb20* WT and cKO mice, verification of *Zbtb20* knockout results. Scale bar, 20 μm.

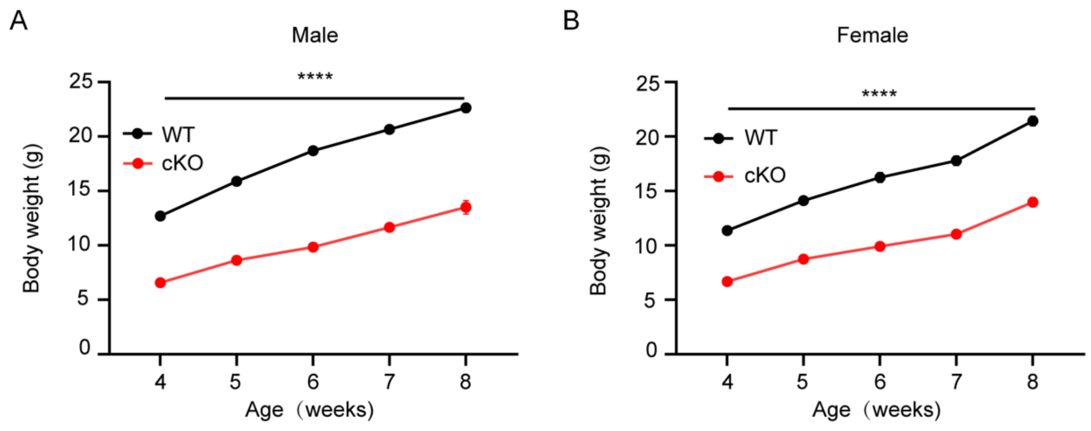

Figure S3. The body weight of *Zbtb20* cKO mice was significantly lower than that of WT mice. (A) Body weight statistics of *Zbtb20* WT and cKO mice aged 4-8 weeks. (B) Body weight statistics of *Zbtb20* WT and cKO female mice aged 4-8 weeks. Data are presented as means ± SEM, ****p < 0.0001, unpaired t-test.

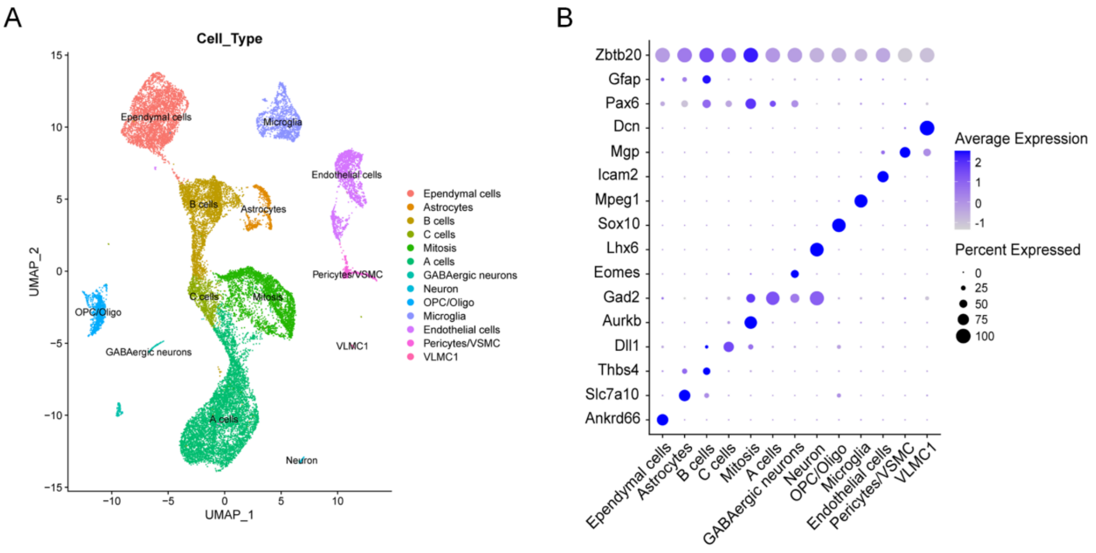

Figure S4. (A) Single-cell clustering analysis of SVZ scRNA-seq data using UMAP plots to identify and visualize cell clusters. (B) Expression patterns of canonical marker genes across SVZ cell types. Dot size indicates the proportion of cells expressing the gene within each cluster, while color intensity reflects expression levels. Zbtb20 is highly expressed in the SVZ, with the highest expression observed in cell lineages exhibiting proliferative capacity.

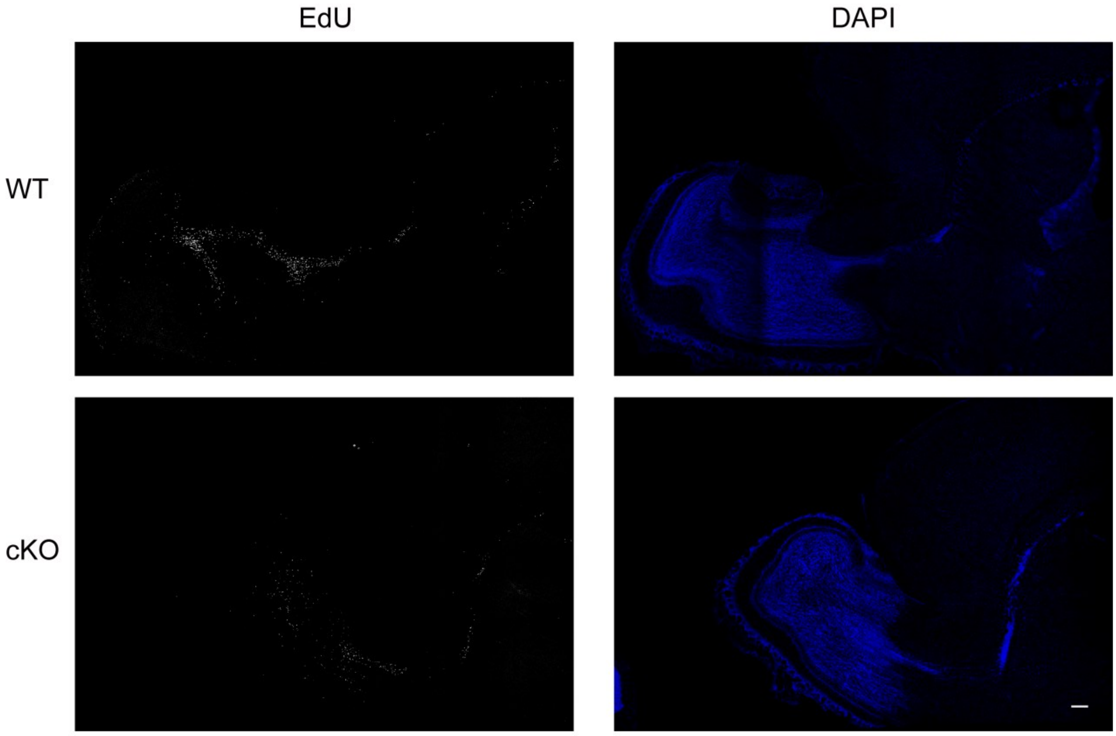

Figure S5. Stem cell migration in the SVZ were affected in *Zbtb20* conditional knockout mice. Immunofluorescence imaging of EdU stained by RMS after EdU injection for 5 days in *Zbtb20* WT and cKO mice at 8 weeks of age. Scale bar, 200 μm.

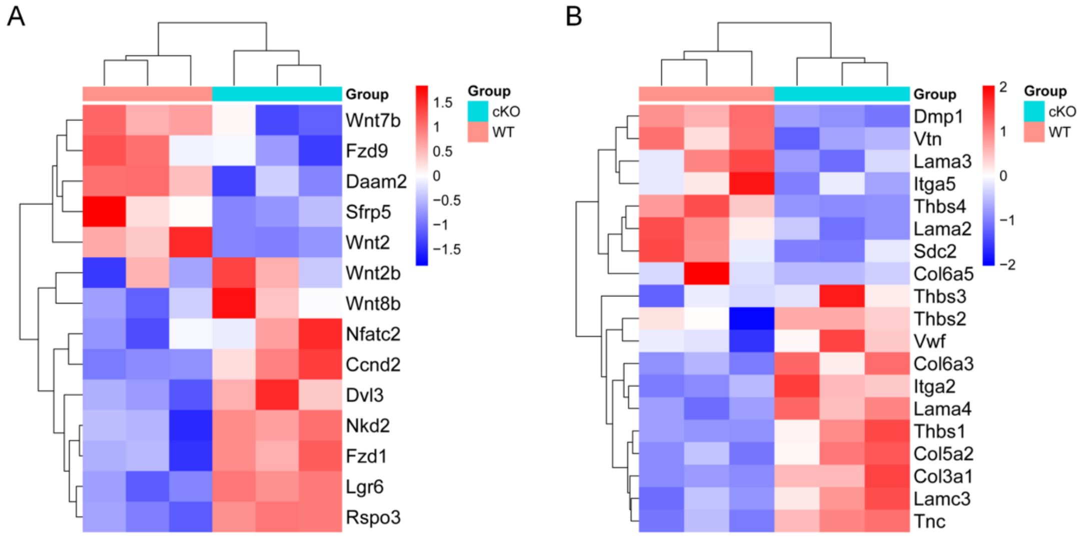

Figure S6. (A) Heat map of differentially expressed Wnt signaling pathpay gene in SVZ brain region of 8-week-old *Zbtb20* WT and cKO mice. (B) Heat map of differentially expressed ECM receptor interaction gene in SVZ brain region of 8-week-old *Zbtb20* WT and cKO mice.

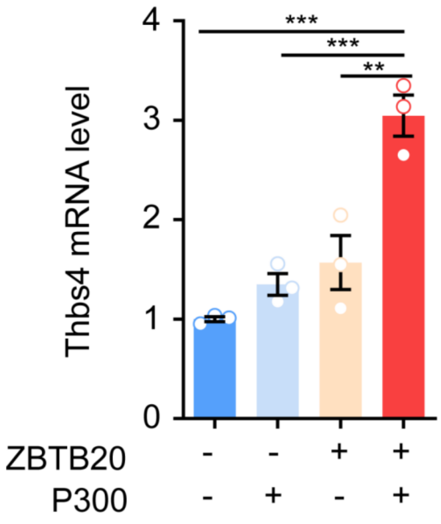

Figure S7. Following transfection of N2a cells with empty vector, P300-myc plasmid, wild-type ZBTB20 plasmid, or co-transfection of P300-myc + wild-type ZBTB20 plasmids, the mRNA expression levels of Thbs4 were analyzed by quantitative PCR (qPCR). Data are presented as means ± SEM, $^{**}p < 0.01$, $^{***}p < 0.001$, one-way ANOVA followed by Tukey's post hoc test.

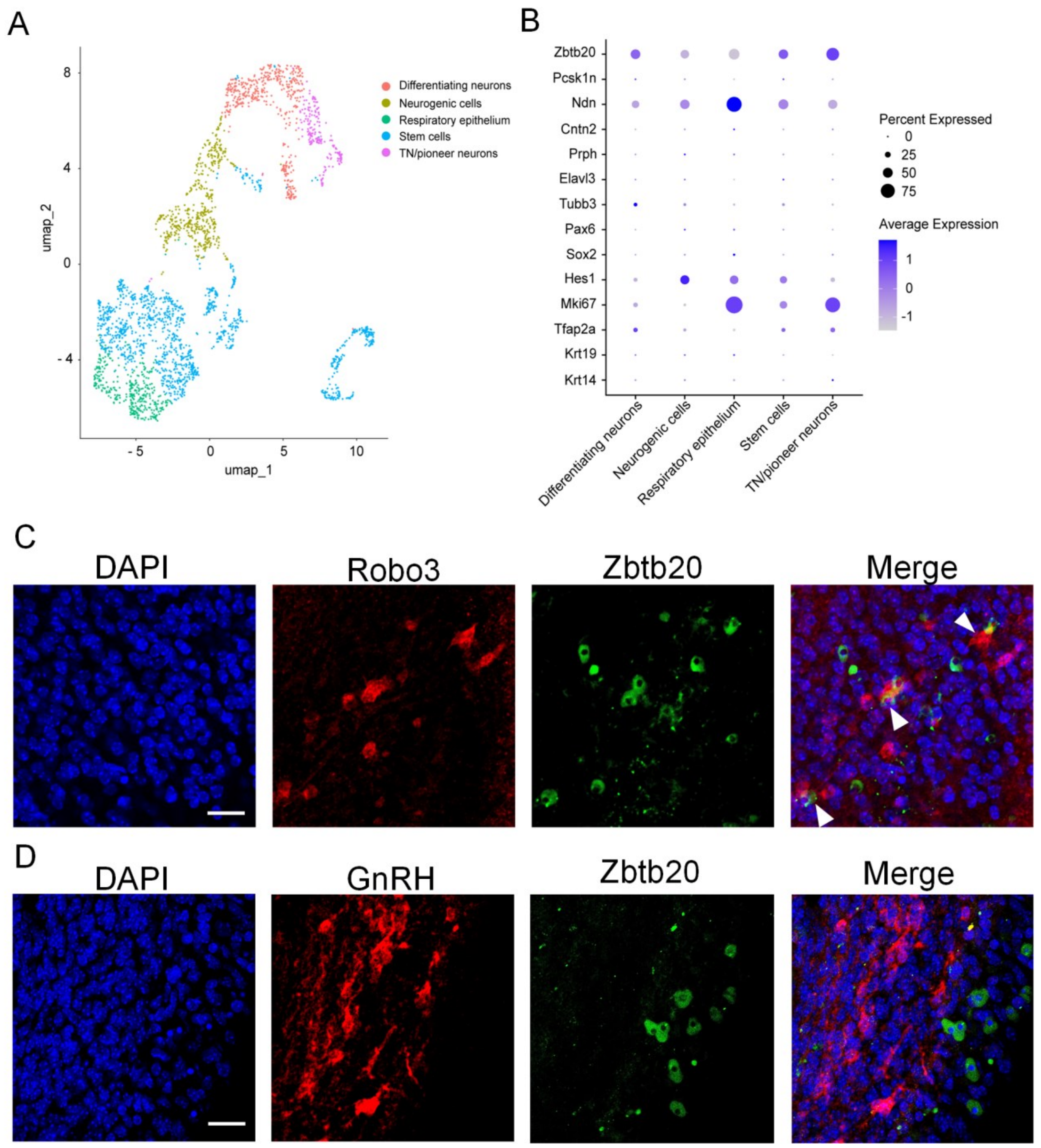

Figure S8. Expression of Zbtb20 in TN and GnRH neurons. (A) Single-cell clustering analysis of scRNA-seq data of whole embryonic mouse noses using UMAP plots to identify and visualize cell clusters. (B) Expression patterns of Zbtb20 and canonical marker genes in different cell types from the embryonic mouse noses. Dot size indicates the proportion of cells expressing the gene within each cluster, while color intensity reflects expression levels. (C) Double immunofluorescence staining of Robo3 and Zbtb20 on mouse noses sections. Scale bar, 20 μm. (D) Double immunofluorescence staining of GnRH and Zbtb20 on mouse noses sections. Scale bar, 20 μm.

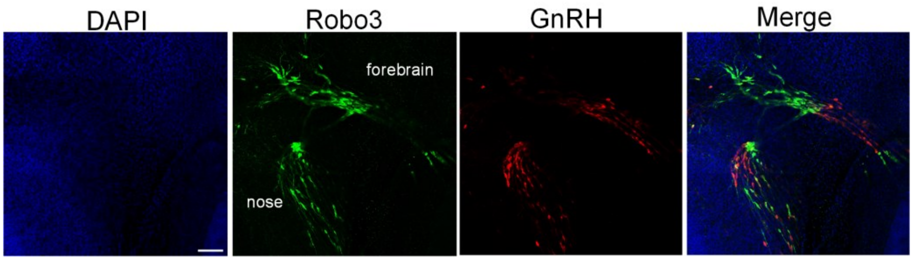

Figure S9. Immunofluorescence staining of Robo3 and GnRH in the embryonic sections (E14.5).Scale bar, 100 μm.

Supplementary Table S1

Clinical and laboratory findings of the CHH family members carrying *ZBTB20* c.C898T: p.R300C

No.	Sex	Dx	Age	LH	FSH	T	E	Bone density	Other phenotypes
I:1	F	Delayed puberty	/	/	/	/	/	low	Hyposmia
II:2	M	Delayed puberty	54	/	/	/	/	low	Hyposmia, right cryptorchidism,
II:6	M	KS	48	0.17	0.87	0.094	11	low	Facial asymmetry, cervical scoliosis, mild cognitive impairment, cryptorchidism
III:1	F	KS	36	0.33	0.79	0.1	15	low	Facial asymmetry, cervical scoliosis, mild cognitive impairment
III:7	M	KS	36	0.01	0.91	0.5	17	low	Facial asymmetry, cervical scoliosis, bilateral cryptorchidism
IV:1	F	KS?	7	/	/	/	/	/	Anosmia, mild cognitive impairment
IV:2	M	KS?	5	/	/	/	/	/	Anosmia, facial asymmetry, bilateral cryptorchidism, micropenis

M, Male; F, Female; Dx, diagnosis; CHH, congenital hypogonadotropic hypogonadism; KS, Kallmann syndrome; Age means the age of diagnosis. Normal adult men reference range for testosterone (T) 1.75-7.81 ng/ml, for Estradiol (E2) <53 pg/ml, for luteinizing hormone (LH) 1.2-8.6 mIU/ml, and for follicle-stimulating hormone (FSH) 1.3-19.3 mIU/ml. Normal adult female reference range for T 0.1-1.5 ng/ml, for E2 31-136 pg/ml, for LH 1.9-12.5 mIU/L, and FSH 1.3-11 mIU/L.

Supplementary Table S2 A virtual CHH gene panel with known and candidate CHH genes

Gene	Chromosome Position	OMIM	Mode of inheritance	PMID
ANOS1	Xp22.31	300836	XLR	11297579
AXL	19q13.2	109135	AD	24476074
CCDC141	2q31.2	616031	AR	28324054
CHD7	8q12.2	608892	AD	29144511
DMXL2	15q21.2	612186	AR	25248098
DUSP6	12q21.33	602748	AD	23643382
FEZF1	7q31.32	613301	AR	25192046
FGF17	8p21.3	603725	AD	23643382
FGF8	10q24.32	600483	AD	18596921
FGFR1	8p11.23	136350	AD	16764984
FLRT3	20p12.1	604808	AD	23643382
FSHB	11p14.1	136530	AR	25071724
GNRH1	8p21.2	152760	AR	19567835
GNRHR	4q13.2	138850	AR	21664415
HESX1	3p14.3	601802	AD	25071724
HS6ST1	2q14.3	604846	AD	21700882
IL17RD	3p14.3	606807	AD	23643382
KISS1	1q32.1	603286	AR	25071724
KISS1R	19p13.3	604161	AR	21880801
KLB	4p14	611135	AR	28754744
LEP	7q32.1	164160	AR	17229951
LEPR	1p31.3	601007	AR	17229951
LHB	19q13.33	152780	AR	25071724
NDN	15q11.2	602117	AR	21543378
NR0B1	Xp21.2	300473	XLR	23295288
NSMF	9q34.3	608137	AD	15362570
OTUD4	4q31.21	611744	AR	23656588
PCSK1	5q15	162150	AR	19707180
PLXNA1	3q21.3	601055	AR	28334861
PNPLA6	19p13.2	603197	AR	24355708
PROK2	3p13	607002	AD	17054399
PROKR2	20p12.3	607123	AD	21037178
RNF216	7p22.1	609948	AR	23656588
SEMA3A	7q21.11	603961	AD	22927827
SEMA3E	7q21.11	608166	AD	25985275
SEMA7A	15q24.1	607961	AR	24522099
SOX10	22q13.1	602229	AD	23643381
SOX2	3q26.33	184429	AD	16932809
SPRY4	5q31.3	607984	AD	23643382
STUB1	16p13.3	607207	AR	24113144
TAC3	12q13.3	162330	AR	20332248

TACR3	4q24	162332	AR	20332248
WDR11	10q26.12	606417	AD	20887964
NDNF	4q27	616506	AD	31883645
GLI3	7p14.1	165240	-	31767679
POLR3B	12q23.3	614366	AR	27512013
NELF	6p21.33	154040	AD	15362570
NOS1	12q24.22	163731	-	36197968
JAG1	20p12.2	601920	AD	36729644
AMH	19p13.3	600957	AR	31291191
AMHR2	12q13.13	600956	AR	31291191
NRP1	10p11.22	602069	-	34636164
NRP2	2q33.3	602070	-	21059704
TCF12	15q21.3	600480	AD, AR?	32620954
PRDM13	6q16.2	616741	AR	34730112
PTCH1	9q22.32	601309	AR	32074614
SOX3	Xq27.1	313430	-	35295983
PLXNB1	3p21.31	601053	-	35170806
NTN1	17p13.1	601614	AD	29202173
DCC	18q21.2	120470	-	29202173
SEMA3F	3p21.31	601124	-	33495532
PLXNA3	Xq28	300022	-	33495532
OTX2	14q22.3	600037	AD	21436260
SRA1	5q31.3	603819	-	27086651
CPEB4	5q35.2	610607	-	38628584
SIN3A	15q24.2	607776	AD	36758531
TUBB3	16q24.3	602661	AD	25559402
TBX3	12q24.21	601621	-	30550377
SCEL	13q22.3	604112	-	31325086
ROBO1	3p12.3	602430	-	31325086
ROBO2	3p12.3	602431	-	31325086
SMCHD1	18p11.32	614982	-	28067909
IGSF10	3q25.1	617351	-	27137492
POGZ	1q21.3	614787	AD	37619992
CCDC103	17q21.31	614677	AR	37108593
SLIT2	4p15.31	603746	-	35797970
ZBTB20	3q13.31	606025	-	27657167
TSHZ1	18q22.3	614427	-	35968788
NHLH2	1p13.1	162361	AR	35066646
CHL1	3p26.3	607416	XLD?	36315353

Supplementary Table S3 The molecular genetic characteristics of the CHH probands with identified *ZBTB20* variants

ID	Gender	Dx	c.HGVS	p.HGVS	Segregation	MAF		ACMG	In silico prediction			
						ALL	EAS		CADD	SIFT	Polyphen2	MutationTaster
L1633	M	KS	ZBTB20:c.172T>C	p.S58P	Maternal	0.00005698	0.000821	U	18.53	T	B	D
			PNPLA6: c.3386G>T	p.G1129V	Maternal	0	0	U	24.7	D	D	D
			PNPLA6: c.3534G>C	p.W1178C	Paternal	0	0	U	31	D	D	D
L1759	M	KS?	ZBTB20:c.1250C>G	p.A417G	Maternal	0.002764	0	LB	12.97	T	B	P
			FGFR1:c.1471C>T	p.Q491X	Maternal	0	0	P	43	D	D	A
			PROKR2:c.533G>C:	p.W178S	Paternal	0.0002051	0.002807	P	31	D	P	D
L1800	M	KS	ZBTB20:c.898C>T	p.R300C	Paternal	0	0	P	35	D	P	D
XH-1	M	KS	ZBTB20:c.1561C>T	p.P521S	NA	0.000007957	0	U	22.1	T	B	D
XH-2	M	KS	ZBTB20:c.1298C>T	p.P433L	NA	0.000003979	0	LP	32	D	P	D
XH-3	M	KS	ZBTB20:c.172T>C	p.S58P	NA	0.00005698	0.000821	U	18.53	T	B	D
XH-4	F	KS	ZBTB20:c.172T>C	p.S58P	NA	0.00005698	0.000821	U	18.53	T	B	D

CHH, congenital hypogonadotropic hypogonadism; M, male; F, female; Dx, diagnosis; KS, Kallmann syndrome; HGVS, Human Genome Variation Society; NA, not available. MAF, minor allele frequency in gnomAD database; EAS, East Asian population in gnomAD; ALL: all populations in gnomAD. ACMG criteria: P, pathogenic; B, benign; U, uncertain significance; LP, likely pathogenic; LB, likely benign. CADD: Combined Annotation Dependent Depletion, the scores ≥ 20 suggests a potentially deleterious variant, ≥ 30 indicates high confidence for damaging effects, and scores below 10-15 are typically considered benign. SIFT: T, tolerated; D, deleterious. PolyPhen-2: B, benign; P, possibly damaging; D, damaging. Mutation Taster: N, polymorphism; D, disease causing; A, disease_causing_automatic; P, polymorphism automatic.

Supplementary Table S4 The information for plasmids used in this study

Plasmid	Vector	Promoter
pGL3-Thbs4-luc	pGL3-Basis	Thbs4 promoter (-2034 to +1)
P300-Myc	pCMV	CMV
ZBTB20-WT	pcDNA3.1	CMV
ZBTB20-c.T172C	pcDNA3.1	CMV
ZBTB20- c.C898T	pcDNA3.1	CMV
ZBTB20- c.C1250G	pcDNA3.1	CMV
ZBTB20- c.C1298T	pcDNA3.1	CMV
ZBTB20- c.C1561T	pcDNA3.1	CMV

Supplementary Table S5 Primers for quantitative PCR of mouse Thbs4 transcript

Primer name	Primer sequence
q-mThbs4-F	GACGGCTATGTCTGTGGAAAGG
q-mThbs4-R	TCCGATGCCATCTCTGTCTGCA

Supplementary Table S6

The primer sequences targeting the *Thbs4* promoter region in ChIP analysis

Primer name	Primer sequence
Thbs4-promoter-P1-F	ATCCCCCATTTGCCAGGAAG
Thbs4-promoter-P1-R	CCCACTGTCCACCTCATTCC
Thbs4-promoter-P2-F	TTGACTGCTGATTCCCCCAC
Thbs4-promoter-P2-R	TCCAACACCCCCGCATTAAA
Thbs4-promoter-P3-F	TCCAATCCCTCTCCACTCCT
Thbs4-promoter-P3-R	ACCTGCCCCTTTTGTGTGTT
Thbs4-promoter-P4-F	ATACTGCCTCACACCACAGC
Thbs4-promoter-P4-R	GAACAGCGACATCTGACCCA
